# Comparison of kinematic parameters of children gait obtained by inverse and direct models

**DOI:** 10.1371/journal.pone.0270423

**Published:** 2022-06-24

**Authors:** Jurgita Ziziene, Kristina Daunoraviciene, Giedre Juskeniene, Juozas Raistenskis

**Affiliations:** 1 Department of Biomechanical Engineering, Vilnius Gediminas Technical University, Vilnius, Lithuania; 2 Faculty of Medicine, Department of Rehabilitation, Physical and Sports Medicine, Health Science Institute, Vilnius University, Vilnius, Lithuania; IRCCS E. Medea, ITALY

## Abstract

The purpose of this study is to compare differences between kinematic parameters of pediatric gait obtained by direct kinematics (DK) (Plug-in-Gait) and inverse kinematics (IK) (AnyBody) models. Seventeen healthy children participated in this study. Both lower extremities were examined using a Vicon 8-camera motion capture system and a force plate. Angles of the hip, knee, and ankle joints were obtained based on DK and IK models, and ranges of motion (ROMs) were identified from them. The standard error of measurement, root-mean-squared error, correlation *r*, and magnitude-phase (MP) metrics were calculated to compare differences between the models’ outcomes. The determined standard error of measurement between ROMs from the DK and IK models ranged from 0.34° to 0.58°. A significant difference was found in the ROMs with the exception of the left hip’s internal/external rotation. The mean RMSE of all joints’ amplitudes exceeded the clinical significance limit and was 13.6 ± 4.0°. The best curve angles matching nature were found in the sagittal plane, where *r* was 0.79 to 0.83 and MP metrics were 0.05 to 0.30. The kinematic parameters of pediatric gait obtained by IK and DK differ significantly. Preferably, all of the results obtained by DK must be validated/verified by IK, in order to achieve a more accurate functional assessment of the individual. Furthermore, the use of IK expands the capabilities of gait analysis and allows for kinetic characterisation.

## Introduction

Walking is a daily physical activity, which is important for everyone, both adults and children. In order to assess various disorders, it is very important to assess gait as accurately as possible. Experimental gait assessment studies are a common practice, performed in specialized gait laboratories using optical or video motion capture systems, inertial sensors, and force plates [1–3]. Kinematic and kinetic parameters are obtained during such gait studies. When studying gait with 3D motion capture systems, computational models are used [4,5], which calculate the joint kinematics according to the appropriate arrangement of markers on the body segments by direct kinematics (DK). In DK, joint kinematic parameters are calculated as Euler angles directly from 3D markers positions [6,7]. However, DK used in gait laboratories does not provide all the necessary information, such as the magnitude of the forces acting on the joints and the forces developed by the individual muscles and their activations. These parameters are very important as they can be used for the clinical planning of surgery or clinical decision-making [8,9], however, they can only be attained through the whole body or the lower limb musculoskeletal models. Musculoskeletal models, in contrast to DK models, calculate joint angles by inverse kinematic (IK) methods, i.e. global optimisation, which aims to minimise the difference between the positions of experimental and modelled markers, while also estimating errors in the marker locations [10]. After performing the IK and obtaining the kinematic parameters, inverse dynamics (ID) are performed, during which the kinetic parameters (such as joint reaction forces, moments, muscle forces, and activations) are obtained. Thus, the accuracy of IK results also determines the ID parameters. So, it is clear that the DK and IK models differ, in terms of technical design, the degree of freedom, the functionality of the joints, and the calculation algorithms implemented there. As a result, we can naturally expect differences in the results obtained.

With the emergence of user–friendly software, such as AnyBody [11] or OpenSim [12], the popularity and application of musculoskeletal (MSK) models in various fields, such as ergonomics [13,14], sports [15,16], and rehabilitation [17,18], are growing rapidly. In addition to various modern experimental gait protocols, which allow direct measurement of motion trajectories, inertial parameters, kinetic or electromyographic (EMG) data, MSK models are increasingly used [8,19,20]. The accuracy of the most used dynamic MSK models of the lower limbs in gait analysis depends on their geometry and the nature of the control signal. The best results are achieved using personalised dynamic models, in terms of the adaptation of the MSK to individual anthropometry [21], and these are controlled by the EMG signal [22–24]. However, if two different multi-body systems, based on human models, use the same anthropometry and the model marker placements are individualised accordingly, then the IK and ID results of these models will be analogous [25–27].

However, the individualised MSK model geometry is only achievable using diagnostic images, which are expensive (magnetic resonance imaging—MRI) and harmful, due to ionising radiation (computerized tomography—CT). Furthermore, they are time-consuming and require large computer resources. In clinical practice, musculoskeletal models of generic geometry (GMSK) are the easiest to use, avoiding the segmentation of human body structures. GMSK models are adapted by scaling according to the height of the subject or the length and weight of the individual segments. It should be noted that the results of GMSK scaling may differ, depending on which scaling method is used in a particular model [28–30]. For example, in the AnyBody ‘Simple Plug-in-Gait’ model, linear specific length-mass scaling with fat percent method is used [30]. Currently, several GMSK models have been developed, based on experimental motion recording data and it should be emphasised that these models have a musculoskeletal model geometry based on adult anatomical structures and are used to determine adult parameters; they are not routinely used for children’s motion analysis [31–34]. In other words, there are only a few works where the adult GMSK model is used to perform the analysis of a child’s gait [35,36]. Although adult GMSK models of the lower extremities have been commonly used to determine muscle function, it remains unclear as to whether such models are suitable for children, because body weight and mass, segment anthropometric size, and other parameters are very important. In addition, musculoskeletal geometry (and other related parameters in both healthy and multi-pathological children) change rapidly as the body grows or disease progresses [37–39]. In pursuance of using existing generic models for children, only comprehensive and accurate validation or comparison analysis will lead to more objective quantitative results. Experimental data, such as EMG and ground reaction forces (GRF), are commonly used to validate musculoskeletal models [40–42]. Data are compared using a variety of statistical and mathematical methods, i.e., correlation coefficients, root mean square error (RMSE), root mean square deviation (RMSD), Magnitude-Phase-Comprehensive (MPC) metrics [43–45], etc., and are calculated to quantify the accuracy and sensitivity of the model. It was determined that the reliability of DK models is moderate to good in adults and children with normal body composition, obese children, children with cerebral palsy, or patients after a stroke [46,47]. Correspondingly, when performing 3D gait analysis by both DK and IK methods, the reliability of the results depends very much on how accurately the anatomical landmarks are determined, where and how markers are placed, human body composition, and so on [48–50]. Therefore, it is important to evaluate what differences occur between the kinematic parameters of the DK and IK models. Recent studies have examined the reliability between DK and IK models in obese children, where it was found that there are no differences in the reliability in joint kinematics between IK (‘gait2392’ OpenSim) and DK (Cleveland Clinical marker set) models [51]. However, large differences in joint kinematics between IK (‘gait2392’, OpenSim) and DK Plug-in-Gait (PiG) models were identified [52], as well as small differences between IK (Human Body Model) and DK PiG [53]. Comparing different DK and IK models yields different results due to the different anatomical models, marker sets, joint constraints, and so on. To the authors’ knowledge, no comparative study has been performed between the DK PiG and IK AnyBody ‘Simple Plug-in-Gait’ models [4,11,54]. Moreover, our work is novel because we studied children’s gait using these models and analysed the results of both legs separately.

The first question before starting this investigation was whether DK models provide accurate enough results, which could be used to assess an individual’s functional condition, since they are widely used in clinical practice. How should they be interpreted and evaluated? In order to be sure of the accuracy and reliability of the further analysis, it is necessary to know how different the kinematic parameters obtained from DK and IK models are.

Therefore, the main objective of this paper is to identify the differences between the most commonly used model, DK PiG, which is still the current benchmark for clinical gait assessment and IK AnyBody ‘Simple Plug-in-Gait’. We aim to deduce whether DK results can be used as indicators of motion in clinical practice or whether they are still insufficient to support clinical decisions. To achieve this goal, we set the following tasks: 1) perform experimental measurements of children’s gait and define kinematics based on the DK PiG model; 2) adjust and scale an adult generic musculoskeletal model in AnyBody software to fit a child and calculate kinematic parameters based on this IK model; and 3) compare the results from both models.

## Materials and methods

### Participants

Only healthy children participated in the study (which was approved by the Regional Bioethics Committee (No. 2020/9-1256-738)) and the consent of both parents was received. The inclusion criteria for enrolling children in the study were: 1) aged four to eleven years; 2) a muscle strength of the lower extremities of not less than 5 points, according to the Lovett scale; 3) ability to understand and follow instructions; and 4) absence of motor disorders affecting gait parameters. Exclusion criteria included: 1) severe visual impairment; 2) concentration and other important behavioural disorders. Thus, based on the inclusion/exclusion criteria, the study included 17 healthy children (11 girls and 6 boys).

### Protocol

**Fig 1:**
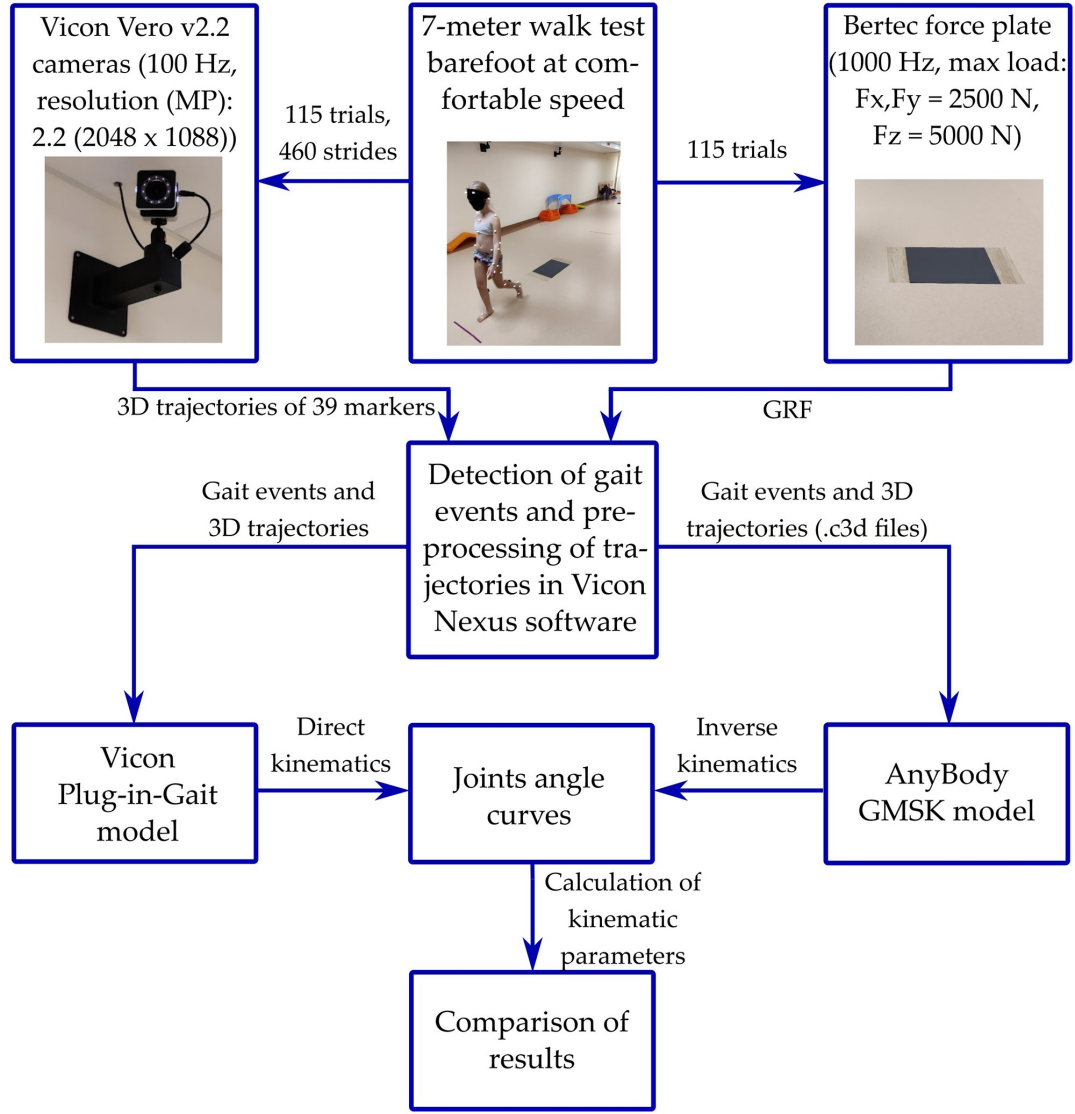
Flowchart of research.

A total of 460 strides were analysed in the following order: (1) the marker trajectories were analysed using Vicon Nexus 2.10.0 software (Vicon Motion Systems Ltd, UK) and the angles of the joints throughout the full gait cycle and the joints’ ranges of motion (ROMs) were calculated, based on the DK PiG model; (2) the marker trajectories from the motion capture system were exported to a.c3d file and loaded into AnyBody software, where the joints angles and joint ROMs were calculated, based on an adult GMSK model, i.e. IK was performed; and (3) parameters of joint ROMs and the angle values between IK AnyBody and DK PiG models were compared. Fig 1 illustrates the experimental setup and data collection process.

For experimental measurements, an eight camera (Vero v2.2) motion capture system (Vicon 100 Hz) was used, and 39 reflective markers were fixed on the subject’s body according to the full-body PiG model marker set [4]. The detection of gait-events was obtained by a Bertec (Bertec Corporation, USA) force plate (4060–07) at a 1000 Hz sampling frequency. A seven-metre walk test was performed at a comfortable walking speed, where the start and the end of the trail were clearly marked. The tasks were repeated at least 10 times for each subject and two strides were analysed from each trial.

### Description of models

The joint angles and ROMs of each stride were calculated based on the full-body DK PiG model and full-body IK AnyBody model. All joints of the lower extremities of the DK PiG model were defined as ball-and-socket joints with three degrees of freedom (DOF) and capable of rotating in the x-y-z axes [4,5].

Individual anthropometric data of all subjects, such as body mass, height, leg length, Asis Trocanter distance, widths of the knee, ankle, elbow, wrist, and hand thickness, were used for the static calibration of the DK PiG model every time.

A ‘Simple Plug-in-Gait’ adult GMSK model [11,54], freely available in the AnyBody software package (v.7.3, AnyBody Technology A/S, Aalborg, Denmark), was used for all simulations, whose full-body kinematic model consists of 39 degrees of freedom (DOF). The lower limbs of this model were obtained from the Twente Lower Extremity Model [55], containing two spherical hip joints (with 3 DOF), two revolute knee joints (1 DOF), and two universal ankle joints (2 DOF). The IK AnyBody model was scaled to match the anthropometry of each subject [56] and the same full-body PiG model marker set was used as in the experimental measurements.

Although both of the models used in our study allowed us to evaluate the kinematic parameters of the whole body, we only assessed the lower limbs. In order to compare results, we examined 10 DOF (5 DOF for each leg) of both models: 3 DOF in the hip joint, 1 DOF in the knee joint, and 1 DOF in the ankle joint (the subtalar joint was locked) avoiding evaluation of the foot inversion/eversion (whereas the fifth metatarsal fracture markers were not used in experimental measurements).

The PiG model uses direct kinematics to calculate joint kinematics and the AnyBody model uses the inverse kinematics method. In addition, the GSMK model in AnyBody uses an optimisation algorithm to overlay the marker trajectories (determined with Vicon Nexus cameras) onto the trajectories of the model markers.

### Data processing and analysis

The pipeline operation was used in Vicon Nexus software, where the data was filtered using the Woltring filter (with a default mean squared error of 10) to ensure smooth trajectories. These smoothed trajectories were later used to calculate kinematic parameters in the DK model and exported to the IK model. In turn, before performing the inverse kinematics in AnyBody software, the default trajectories were filtered by a 4th order zero-lag low-pass Butterworth filter with a 5 Hz cut-off frequency. Also, a 4th order zero-lag low-pass digital Butterworth filter with 6 Hz cut-off frequency was used for both model outputs (joint angles). Data obtained from DK PiG and IK Anybody models were exported and subsequently processed using the MatlabR2020b software (MathWorks Inc, USA). A total of 115 trials and 230 strides were further processed for each leg. The kinematic parameters compared in this work were: hip flexion/extension (F/E), hip abduction/adduction (Ab/Ad), hip internal/external rotation (IR/ER), knee flexion/extension (F/E) and ankle dorsiflexion/plantarflexion (D/P). Firstly, joint angle curves were cropped from heel-to-heel contact to strides according to the force plate data and normalised to the full gait cycle. Secondly, the ROMs of the joints were the differences between the maximum and minimum point of the angles calculated for DK PiG and IK AnyBody model results. The interquartile range (IQR), with 25th and 75th percentiles, and the standard error of measurement (SEM) between models, were calculated for ROMs. The values of joint angles predicted in the IK AnyBody model were compared with the DK PiG model values and the accuracy of the results was evaluated. The differences between joints amplitudes were quantified using the root-mean-squared error (RMSE), which was calculated by a point-to-point approach.

A clinically significant quantity, which should be at least 5°, was determined for the SEM and the RMSE values [46].

The covariance between mean values of the DK PiG and IK AnyBody joint angles was estimated by calculating the Pearson correlation coefficient (*r*) (*p < 0*.*05*), wherein if the p-value is less than or equal to the significance level, then the correlation is different from 0, i.e. the correlation is statistically significant. The absolute values of the coefficient can be classified as: *r* ≤ 0.35 weak correlation; 0.36 ≤ *r* ≤ 0.67 moderate correlation; 0.68 ≤ *r* < 0.9 high correlation; and *r* ≥ 0.9 very high correlation [57].

Subsequently, the magnitude (M) and phase (P) metrics between DK and IK models’ joint angle curve values were calculated according to the Sprague-Geers methodology, based on point-to-point comparison [58,59]:

$$M=\sqrt{\frac{\vartheta_{cc}}{\vartheta_{mm}}}\;-1$$ (1)

$$P=\;\frac{1}{\pi }cos^{-1}\frac{\vartheta_{cm}}{\sqrt{\vartheta_{cc}\vartheta_{mm}}}$$ (2)

where *ϑ*_*cc*_, *ϑ*_*mm*_ and *ϑ*_*cm*_ are time integrals, and they were calculated according to:

$$\vartheta_{cc}=\;{\left(t_{1}-t_{2}\right)}^{-1}\int_{t_{1}}^{t_{2}}c^{2}\left(t\right)dt,$$ (3)

$$\vartheta_{mm}=\;{\left(t_{1}-t_{2}\right)}^{-1}\int_{t_{1}}^{t_{2}}m^{2}\left(t\right)dt,$$ (4)

$$\vartheta_{cm}=\;{\left(t_{1}-t_{2}\right)}^{-1}\int_{t_{1}}^{t_{2}}c\left(t\right)m\left(t\right)dt,$$ (5)

where *m*—are the values of the DK PiG joint angles; *c*—are the values of the IK AnyBody joint angles, and *t*_*1*_ ≤ *t* ≤ *t*_*2*_ is the time span of the joint angles. Metrics is a statistical method that determines the difference between curves both between joint amplitude values (y-axis as offset) and time values (x-axis as delay). Metrics between models were calculated in two ways: 1) between the averaged values of all strides; 2) between values of joint amplitudes in each stride; and then the MP metrics data were averaged. M is not sensitive to phase discrepancies, while P is not sensitive to magnitude differences. For kinematic data comparison, the following M and P metrics evaluation levels were set: 0–0.2 ‘really good matching’; 0.2–0.3 ‘fair matching’; 0.3–0.4 ‘rather poor matching’; 0.4–1.0 ‘poor matching’; and more than 1 represents ‘no matching’ [59]. Negative values of M mean that the IK AnyBody curves have lower values than DK PiG and positive values indicate that the curves have higher values.

SEM, RMSE, correlation coefficient, and MP metrics of angle values were calculated without removing the offset and time delay.

Statistical power analysis was performed with G.Power 3.1 in order to estimate sample size. It was determined that a total of 16 samples would be needed to detect a difference in the results obtained by different models. Shapiro-Wilk’s Normality Test was used to check the normality of the data [60]. Not normally distributed data were presented as the median (median absolute deviation (MAD) with IQR) and normally distributed data as the mean ± standard deviation (SD). ROMs results were statistically evaluated between DK PiG and IK AnyBody model values. A non-parametric, two-tailed Wilcoxon signed-rank test (*p < 0*.*05*) was used for not normally distributed data. Statistical analysis was performed using the MatlabR2019b software (MathWorks Inc, USA).

## Results

The average age ± standard deviation (SD) of 17 children who participated in the study was 7.88 ± 1.97 years, with 11 girls (64.71%) and six boys (35.29%); the average height was 1.31 ± 0.11 m (mean ± SD), and body mass was 28.75 ± 7.41 kg.

Table 1 displays the median ROMs (MAD), IQR, and SEM of the DK PiG and IK AnyBody model results, which were determined for each step of all experimental trials.

**Table 1:** DK PiG and IK AnyBody modelled ROMs of joints, their IQR intervals, and SEM values (n = 230).

Parameters			Hip F/E	Hip Ad/Ab	Hip IR/ER	Knee F/E	Ankle D/P
**PiG ROM,°**	Right	median (mad)	**43.9 (3.9)**	**12.5 (2.9)**	**26.6 (7.5)**	**52.2 (6.9)**	**31.7 (5.9)**
		IQR	41.2–47.4	10.3–15.1	20.7–33.0	43.8–56.9	26.7–35.9
	Left	median (mad)	**43.9 (3.5)**	**12.3 (3.2)**	19.2 (6.9)	**53.5 (4.8)**	**31.0 (6.4)**
		IQR	41.6–46.8	10.5–15.8	14.3–26.3	49.8–57.2	26.2–36.9
**GMSK ROM,°**	Right	median (mad)	**47.5 (9.6)**	**23.8 (7.7)**	**15.6 (4.2)**	**66.3 (8.9)**	**27.7 (5.2)**
		IQR	40.5–57.1	16.6–30.9	12.2–19.2	58.6–72.9	23.1–32.4
	Left	median (mad)	**57.8 (6.8)**	**23.2 (6.3)**	19.9 (6.4)	**55.8 (7.4)**	**29.2 (5.4)**
		IQR	52.8–64.3	18.6–28.8	16.3–25.5	50.8–64.4	24.1–33.8
**SEM, °**	Right		0.44	0.43	0.46	0.58	0.34
	Left		0.47	0.40	0.39	0.38	0.35

**bold** indicates a significant difference (*p < 0*.*05*) between the same body side of DK PiG and IK AnyBody ROMs.

The results show that there is a significant difference between ROMs obtained by both models with an exception in the left leg hip IR/ER (*p < 0*.*05*). The SEM between models’ ROMs in all motions and planes does not exceed the clinical significance limit, i.e. less than 5°.

**Fig 2:**
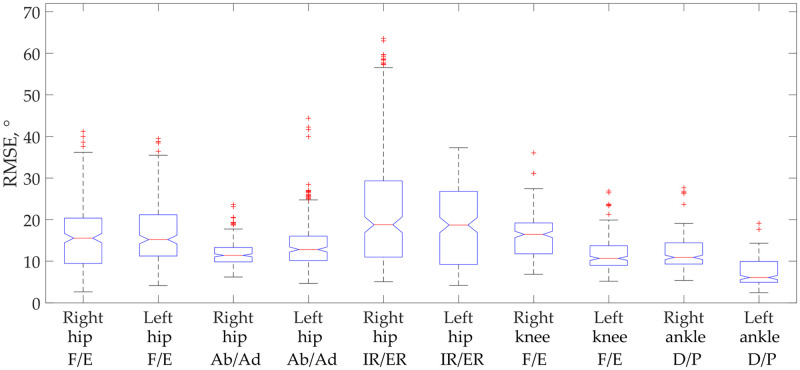
RMSE box plots between DK PiG and IK AnyBody angle values of all joint movements.

The mean RMSE values with IQR intervals at each point of the gait cycle are shown in Fig 2. The lowest mean RMSE value (6.1 (2.6)) was observed in the left ankle D/P and the highest (18.8 (12.3)) in the right hip IR/ER angles.

**Fig 3:**
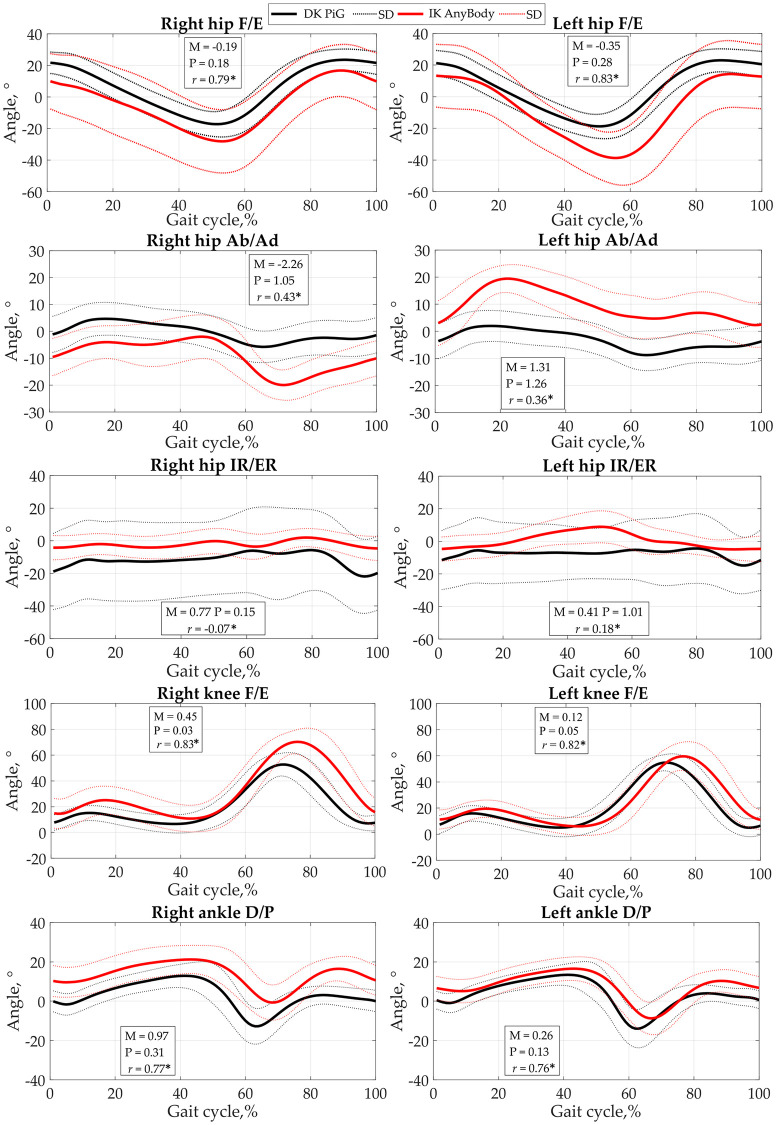
Average of DK PiG and IK AnyBody joint angle curves ± SD during the gait cycle. M is the magnitude error, P is the phase error (taken from the Sprague-Geers metrics), and *r* is the Pearson correlation coefficient (*—the correlation is statistically significant).

The averages of the DK PiG and IK AnyBody model angle curves, their MP metrics, and the correlation coefficients, are shown in Fig 3. All obtained correlations were statistically significant. A high correlation was determined for all movements performed in the sagittal plane, i.e. hip F/L, knee F/L, and ankle D/P. A moderate correlation was indicated in the frontal plane (hip Ab/Ad) and a weak correlation was observed in the transverse plane (hip IR/ER).

The comparison level in the right hip F/E and both sides of the knee F/E was ‘really good’ (MP < 0.2) in all MP metrics. In the left hip F/E, it was noticed ‘fair phase’ and a ‘rather poor’ magnitude match. Hip IR/ER congruence of magnitude ranges from ‘rather poor’ in the left to ‘poor’ in the right, although the compliance of the phase is ‘really good’ in the right but does not match in the left (P > 1.00). Ankle D/P left phase falls into the ‘really good’ level and the right phase falls into the ‘fair’ level. Ankle D/P left magnitude displayed the ‘fair’ level, and right magnitude disclosed a ‘poor’ level. Only the MP parameters of hip Ab/Ad angles do not match (M and P of more than 1).

A more detailed analysis of MP metrics, separately comparing the curve values of each stride, is presented in Table 2.

**Table 2:** Sprague-Geers metrics for the comparison between each stride of DK PiG and IK AnyBody joint angle curve values (n = 230).

Parameters			Hip F/E	Hip Ad/Ab	Hip IR/ER	Knee F/E	Ankle D/P
M	Right	median (mad)	-0.20 (0.25)	-1.53 (0.95)	0.39 (0.23)	0.18 (0.15)	0.30 (0.17)
		IQR	-0.40–0.06	-0.77-(-2.38)	0.18–0.54	0.03–0.35	0.20–0.49
	Left	median (mad)	-0.28 (0.26)	1.51 (0.94)	0.31 (0.20)	0.07 (0.17)	0.15 (0.24)
		IQR	-0.44-(-0.08)	0.63–2.27	0.12–0.41	-0.04–0.26	0.01–0.27
P	Right	median (mad)	0.19 (0.21)	0.73 (0.35)	0.84 (0.53)	0.05 (0.03)	0.28 (0.19)
		IQR	0.08–0.48	0.40–1.04	0.27–1.40	0.03–0.06	0.18–0.53
	Left	median (mad)	0.22 (0.21)	0.89 (0.39)	0.81 (0.47)	0.06 (0.02)	0.14 (0.13)
		IQR	0.11–0.46	0.53–1.31	0.42–1.40	0.05–0.07	0.09–0.29

Comparison levels: green, really good matching; yellow, getting fair matching; orange, rather poor matching; pink, poor matching; and red, not matching.

Examining the MP metrics for each step, some of the results differ from the averages obtained in Fig 3. A total of 10 magnitudes and 10 phase metrics were calculated for both sides of the body. It has been observed that eight metrics (four magnitudes and four phases) even fall within the range where matching is ‘really good’, this is a total of 40% of all values. Also, four metrics (two magnitudes and two phases) fall within the limits of ‘fair’ level, or 20% of all values, and two magnitude metrics fall within the ‘rather poor’ level, accounting for 10% of all values. A total of six metrics (two magnitudes and four phases) fall within the limits that are considered to be ‘poor’ matching and ‘not matched’, comprising 30% of all values.

## Discussion

The aim of this study is to determine the differences in the kinematic parameters of pediatric gait between models based on different calculation methods, i.e. direct and inverse kinematics. It can be seen that DK models are often used to assess movement in the gait laboratory, in clinical practice. They provide the kinematic parameters of the motion, which are the initial decision data, however, the clinical practice is not limited to kinematic indicators. Consequently, even if it is enough to use only the DK model for the calculation of kinematic parameters, then for kinetic parameters it will be necessary to use MSK. Kinetic parameters are required when planning clinical procedures or interventions, adapting measures, or simply making decisions. However, the accuracy of inverse dynamics indicators depends entirely on the obtained kinematics. We emphasise that kinematic parameters are a significant part of clinical evaluation, so it is very important to correctly interpret and evaluate the results. We believe that our research will contribute to the application of the models in practice.

In analysing the results obtained, we want to draw attention to the following facts. Comparing our SEM results of ROMs with those obtained by other studies (direct vs. inverse kinematics), we observed that Horsak et al. [51] found clinically reliable results in all the planes of all the joints examined. Flux et al. [53] only found reliable results in the knee and hip F/E but unreliable hip IR/ER results. Our results were clinically reliable in all ROMs of the movements examined, as the SEM did not exceed 5°. Moreover, ROMs results obtained by the IK AnyBody showed the same trends as in Horsak et al., with the hip Ab/Ad values are being higher than in DK PiG, but hip IR/ER and ankle D/P movement values being less than in DK PiG [51]. The trend for hip F/E and knee F/E is different because we obtained larger ROMs. Flux et al. [53] found that the ROM of hip F/E, and knee F/E is higher, and hip Ad/Ab, hip IR/ER, and ankle D/P are smaller in the inverse kinematics model than in the DK PiG. Our results only confirmed those previously described in other works, i.e. they revealed the same trends (except for hip IR/ER). It has been observed that a good fit of these kinematic parameters is only obtained by using individual geometry models [52]. However, it is not possible to unambiguously compare the results from all of this research due to the different methodologies and different reliability and correlation results. Our study demonstrated a mean RMSE = 13.6 ± 4.0°, while the mean RMSD of all joint angles, presented by Kainz et al, was 6.8 ± 5.0° [52] and, accordingly, Horsak et al. demonstrated that the SEM(averaged across the minimum, maximum and ROMs) was below 3° (range: 1.6–3.0°) [51]. Only Flux et al. set RMSE values over a complete stride that were 11.8°, which was close to our results. We believe that to reduce RMSE values, the offset in the curves should be eliminated, as did Flux et al. [53]. However, we found that the offset and delay differ not only for different joints but even for different subjects. We examined the hip joint in sagittal, frontal, and transverse planes and our results confirmed the findings of other researchers [52,53], i.e. that direct and inverse dynamics models correlate the best in the sagittal plane.

In the reviewed literature, we did not find the results of MP metrics that could be used as parameters for the comparison of joint angle values. However, such metrics are used to validate ground reaction forces (measured vs predicted [45]), as well as for predicted muscle forces with EMG envelopes [43] and other areas [61]. Calculating the metrics between the mean curves of joint angles resulted in worse results than calculating such metrics between each curve separately. Therefore, when comparing the results, we would recommend evaluating the individual curves rather than their averages. Assuming that the properly compared results are those that fall at least to the ‘fair’ comparison level, we are glad to present a 60% matching of our results. In addition, we observed that, in the IK AnyBody model, joint angle curves are most accurately calculated in the sagittal plane. This is confirmed, not only by the MP metrics, but also by the RMSE value determined in other planes. Other scientists have observed such trends. Robinson et al. [62] observed that the largest differences in knee joint amplitudes occur in the frontal plane, although both modelling approaches were similar in their classification of injury risk. Kainz et al. found that the highest RMSD of 11 ± 6° is in hip IR/ER, which is related to our results, since the highest RMSE values of 18.7 (7.9° and 18.8 (12.3° were observed in the same joint movement. However, this can be explained by the fact that, even when conducting gait studies in different laboratories, the same tendency is observed [63,64].

Summarising the results obtained, we singled out two factors that were most likely to influence the results. Firstly, we assumed that the differences between the curves are due to scaling errors, that are observed even when scaling MSKs in children of individual geometry [28]. The results may also have been influenced by the fact, that when scaling, either the joint centre locations are not at the same anatomical point of the segments, or the segment’s anatomy is different [29]. In addition, it can be argued that in movements performed in different planes, scaling has different effects. Secondly, the differences in kinematic parameters between the models may have been due to the different computational methods used in direct and inverse kinematics. Other authors have also found differences arising between direct and inverse kinematics [52,53,62].

Future research should focus on comparing the reliability and accuracy of MSK models with the DK PiG model, which is still the current standard for clinical gait assessment. Thus, the main objective of this study was to identify the differences between direct kinematics Plug-in-Gait vs inverse kinematics AnyBody models. It should be mentioned that only several works compare the kinematic parameters of different models [35,51–53], because model-based gait analysis or model validation on muscle function and related parameters are usually performed by inverse dynamics [40–42,65–68]. Furthermore, we did not find a comparison of the kinematic parameters obtained by the DK PiG and the IK AnyBody models used in our study. Moreover, we used different estimation methods to evaluate the differences in the models, which allowed us to reveal completely distinct properties of the outcomes. We, like most, used SEM, RMSE, and correlation analysis, which allowed us to determine the systematic errors, and the strength of the relationship of the results, respectively. We also used MP metrics to identify curve-type overlaps, which is not common in this type of study. We also stand out from other works because we took the challenge to use both models to analyse children’s gait, a practice that is particularly rare. In addition, both legs were examined separately, which allowed us to assess changes on both sides of the body more accurately.

We believe that research in this field needs to continue but its most important limitations should be considered. Firstly, our study was only performed on healthy children and so children with a pathological gait may present different results. Secondly, a broad age group of children was taken and assessed as a whole. However, we believe that, for children of different ages, these kinematic differences might vary, i.e. they could be greater for younger children. The reason for this could be the higher scaling errors in the generic adult musculoskeletal model due to large differences in anthropometrics between children and adults [28,69].

These identified differences between the results obtained from the models must be taken very seriously, especially in the gait from DK. Clinical judgments should be weighted and errors in the results evaluated accordingly.

## Conclusions

Summarizing the direct and inverse kinematics results of our study, it can be stated that the smallest differences were observed in ROMs of hip F/E, hip Ab/Ad, hip IR/ER, knee F/E, and ankle F/E, whereas the established SEM is in the range of 0.34° to 0.58°, which is less than the clinically significant limit of 5°. Among the models, the high correlation and MP metrics observed in the sagittal plane of the hip, knee, and ankle movements fall into really good/getting fair matching levels, which means a curve-like matching. Unfortunately, large differences in motion angles were observed at different points in the gait cycle, as the RMSE values were higher than the clinical significance limit. DK models available in measurement systems are not so accurate that their results can be used to assess an individual’s functional condition and make further decisions. As primary indicators, these results are appropriate, but they need to be interpreted responsibly and with knowledge of real, possible errors.

## Supporting information

S1 Dataset (XLSX)
